# Gastrointestinal symptom severity and progression in systemic sclerosis

**DOI:** 10.1093/rheumatology/keac118

**Published:** 2022-03-03

**Authors:** Nina M van Leeuwen, Maaike Boonstra, Håvard Fretheim, Cathrine Brunborg, Øyvind Midtvedt, Torhild Garen, Øyvind Molberg, Tom W J Huizinga, Jeska K de Vries-Bouwstra, Anna-Maria Hoffman-Vold

**Affiliations:** Department of Rheumatology, Leiden University Medical Center, Leiden, The Netherlands; Department of Rheumatology, Leiden University Medical Center, Leiden, The Netherlands; Department of Rheumatology; Oslo Centre for Biostatistics and Epidemiology, Research Support Services, Oslo University Hospital; Department of Rheumatology; Department of Rheumatology; Department of Rheumatology; Institute of Clinical Medicine, University of Oslo, Oslo, Norway; Department of Rheumatology, Leiden University Medical Center, Leiden, The Netherlands; Department of Rheumatology, Leiden University Medical Center, Leiden, The Netherlands; Department of Rheumatology; Institute of Clinical Medicine, University of Oslo, Oslo, Norway

**Keywords:** SSc, gastrointestinal symptoms

## Abstract

**Objectives:**

To evaluate the severity and evolution of patient-reported gastrointestinal tract (GIT) symptoms in systemic sclerosis (SSc) patients, assess predictive factors for progression and determine the impact of standard of care treatment.

**Methods:**

SSc patients from the Leiden and Oslo cohorts were included. We assessed clinical data and patient-reported GIT symptoms measured by the validated University of California, Los-Angeles Gastrointestinal-tract (UCLA-GIT) score at baseline and annually. GIT severity and progression was determined. Logistic regression was applied to identify risk factors associated with baseline GIT symptom severity. Linear mixed-effect models were applied to assess progression in GIT symptom burden and to identify predictive factors. We repeated all analysis in patients with early disease (inception cohort) to exclude the effect of longstanding disease and increase insights in development of GIT symptom burden early in the disease course.

**Results:**

We included 834 SSc patients with baseline UCLA GIT scores, 454 from Leiden and 380 from Oslo. In the total cohort, 28% reported moderate-severe GIT symptoms at baseline, with increased risk for severity conferred by ACA, smoking and corticosteroid use, while use of calcium channel blockers appeared protective. In the inception cohort, 23% reported moderate-severe GIT symptoms at baseline, with increased risk for females and with smoking. Over time, symptom burden increased mainly for reflux/bloating. Female sex and ACA predicted GIT symptom progression.

**Conclusion:**

High GIT symptom burden is present early in SSc disease course. Both for prevalence and for progression of GIT symptom burden, female sex and smoking were identified as risk factors.

Rheumatology key messages

- At time of SSc diagnosis, many patients report severe reflux and distension/bloating symptoms.
- Gastrointestinal tract (GIT) symptom burden progresses over time, with highest progression rates for reflux and distension/bloating.
- Female sex, presence of anti-centromere antibody and smoking are associated with GIT symptom burden.

## Introduction

Second to skin, the gastrointestinal tract (GIT) is the most commonly afflicted organ system in SSc [1]. All segments of the gastrointestinal tract can be affected resulting in dysmotility and hypomotility of the oesophagus, the small intestine and the colon with possible life-threatening complications [2]. GIT symptoms have negative impact on quality of life and severe GIT involvement associates with high mortality [3–8]. Molecular mechanisms underlying GIT involvement in SSc are not clear, but the prevailing view is that immune-mediated inﬂammatory processes and progressive vascular abnormalities contribute to fibrotic changes of the bowel wall leading to disturbed intestinal blood ﬂow, poor microcirculation and altered contractility [9–12].

Currently, the approved treatment options for GIT involvement in SSc are limited, but this may, at least partially, relate to the fact that very few intervention studies have addressed GIT-related outcome measures. Studies that have been performed have focussed on SSc patients with advanced GIT disease [2, 13–15].

Knowledge on how SSc-related GIT symptoms develop over time is in general limited. Additionally, little is known about factors that can predict GIT symptom worsening. To target fibrotic and vascular disease complications, SSc patients are frequently treated with immune-modulating and/or vasodilating drugs, which have been reported to have GIT side effects [16–22]. From the European Scleroderma Observational Study we know that patients with recent-onset diffuse cutaneous SSc (dcSSc), the most severe form of SSc, have increasing cumulative incidence of GIT symptoms over 24 months. This increase did not appear to be influenced by the immune-modulating therapies used by the patients [23]. However, it is currently unknown whether these symptoms continue to worsen. Also, the European Scleroderma Observational Study only included patients with dcSSc, while GIT symptoms are also highly prevalent among patients with lcSSc [24].

More high-quality evidence on the severity and course of GIT symptoms in the general SSc population is needed to be able to address everyday clinical challenges. Therefore, we took advantage of two large, prospective SSc cohorts that include annual standardized recording of GIT symptoms, and we set out to answer the following questions: (i) How is the burden of GIT disease among SSc patients? (ii) Which disease characteristics impact on GIT symptom burden in SSc? (iii) How does GIT symptom burden develop over time in SSc? (iv) Can we identify predictors for increasing GIT symptom burden over time in SSc? and (v) Does immunomodulatory treatment impact on development of GIT burden over time?

To answer these questions, we evaluated patient-reported GIT symptoms at baseline and annually, during follow-up, determined predictive factors for GIT symptom progression and assessed associations between standard of care treatment and GIT symptoms.

## Method

### SSc patient cohorts

All SSc patients from prospective, observational SSc cohorts followed at the Leiden University Medical Center and Oslo University Hospital were included for the current study; if they (i) fulfilled the 2013 American College of Rheumatology classification criteria for SSc and (ii) had at least one UCLA GIT score [25]. Clinical data were retrieved from the research databases ‘Combined Care in Systemic Sclerosis approved by the local Ethics Committee P09.003/SH/sh’ in Leiden and the ‘Norwegian systemic CTD and vasculitis registry (NOSVAR) approved by the Regional Committee for Medical&Health Research Ethics South East Norway; 2016–119’ in Oslo, and supplemented with data from electronic patient files [26, 27]. All study patients with disease duration <24 months from disease onset, defined as first non-Raynaud symptom, were also included in the study inception cohort, allowing for separate analyses of GIT symptom early in the disease course, and the effect of standard of care treatment on GIT symptom severity and progression. The cohort study was designed in accordance with the ethical principles of the Declaration of Helsinki. All patients gave written informed consent.

### Patient-reported GIT symptoms

In both SSc centres we started to collect the validated patient reported outcome UCLA GIT 2.0 in 2013 on an annual basis to assess GIT symptoms together with registration in the hospital databases [28]. The UCLA GIT 2.0 questionnaire is a seven-item scale including reflux, distention/bloating, diarrhoea, faecal soilage, constipation, emotional well-being and social functioning capturing SSc-related GIT symptoms and their severity based on the frequency of occurrence. All scales are scored from 0 (better) to 3 (worse) except the diarrhoea and constipation scales (ranges are 0–2 and 0–2.5). The total UCLA GIT score is the sum of all scales (except constipation) and ranges from 0.00–2.83 providing an estimation of the severity of GIT involvement [28, 29] (Supplementary Data S1, available at *Rheumatology* online). To evaluate progression of GIT involvement, the reported and validated minimal clinical important differences (MCID) in the UCLA GIT score between two time points (yearly visits) were assessed (MCID values can be found in Supplementary Data S1, available at *Rheumatology* online) [30].

### Clinical characteristics

The included baseline variables were selected based on reports from literature and expert opinion (Supplementary Data S2 for included variables, available at *Rheumatology* online). As SSc is a multiorgan disease [8], general SSc disease severity at baseline was defined based on a composite score based on individual items that are all validated. Patients were classified as having severe SSc disease in case of presence of one or more of the following: interstitial lung disease (ILD), defined by presence of lung fibrosis on high resolution CT and a forced vital capacity (FVC) <70%; pulmonary arterial hypertension (PAH) with mean pulmonary arteria pressure ≥25 mmHg by right heart catheterization; scleroderma renal crisis; digital ulcers; and/or severe skin involvement defined as modified Rodnan Skin Score (mRSS) >15 [31]. Information about standard of care treatment for SSc was collected and included immunomodulatory drugs for any indication (cyclophosphamide, methotrexate, MMF, azathioprine, corticosteroids, hydroxychloroquine), vasodilating drugs [calcium channel blocker (CCB), angiotensin-converting-enzyme inhibitors (ACE inhibitors), endothelin receptor antagonist (ET-1 inhibitors), phosphodiesterase 5 inhibitor (PDE-5 inhibitor), prostacyclin analogue], and specific GIT medications (proton pump inhibitor, H_2_ antagonist). Use of immunomodulatory medication was collected at every visit, and at each time of completing the UCLA GIT questionnaire. Vasodilatory and GIT drugs were evaluated as ever used. Detailed explanation on organ involvement screening can be found in Supplementary Data S3, available at *Rheumatology* online.

### Statistical analysis

Statistical analyses were performed on SPSS version 25 and STATA version 15. Both the total cohort and the inception cohort were analysed, the inception cohort (disease duration since first non-Raynaud symptom <24 months) was analysed to increase insights in development of GIT symptom burden over time.

To address severity of GIT symptoms among SSc patients, we used descriptive analysis (numbers and percentages). To identify disease characteristics that associate with GIT disease, we used ordinal logistic regressions and described the odds ratios (OR) and the corresponding 95% CI. In this analysis we adjusted for disease severity at baseline as a possible confounder on GIT symptoms, using the composite variable reflecting skin/lung/kidney involvement and PAH (detailed explanation on generating models can be found in Supplementary Data S4, available at *Rheumatology* online). To assess whether specific organ manifestations associate with GIT symptom severity, we applied binary logistic regression analyses. To assess changes in GIT symptom burden over time and to identify possible predictors for worsening of GIT symptoms, linear mixed-effect models were used. Time and risk factors were fixed effects in the analysis. Interaction effects between time and fixed factors were checked. All models included random intercept and slope, and an unstructured correlation matrix was used. To better understand the impact of immunomodulatory treatment on GIT symptoms, we determined GIT symptom progression in patients naïve for immunomodulatory treatment and in treatment exposed patients. For all analyses, Bonferroni–Holm correction was applied to adjust for multiple testing (indicated with * in all tables and Supplementary file, available at *Rheumatology* online).

## Results

### Patient populations

The total study cohort included 834 SSc patients. Demographics and clinical characteristics at baseline were comparable in patients from the Leiden University Medical Center and Oslo University Hospital cohorts (Supplementary Table S1, available at *Rheumatology* online). From the total cohort, 236 patients had disease duration <24 months and these were included in study inception cohort (Table 1).

**Table 1:** Baseline demographic of the total systemic sclerosis cohort, and for the inception cohort

	Total cohort*n* = 834	Inception cohort*n* = 236	*P*
Demographic			
Female, *n* (%)	687 (82)	180 (76)	0.57
Age, mean (s.d.), years	55 (14)	54 (13)	0.91
SSc disease duration at inclusion, median (IQR)	5.9 (1.7–11.9)	0.7 (0.3–1.2)	<0.001
Smoking, ever *n* (%)	420 (50)	122 (62)	0.32
Organ involvement			
Diffuse cutaneous SSc, *n* (%)	186 (22)	67 (28)	0.24
Severe skin involvement, *n* (%)	96 (12)	43 (19)	0.32
Myositis, *n* (%)	51 (6)	18 (8)	0.46
DLCO% <60% of predicted, *n* (%)	267 (33)	71 (31)	0.75
FVC% <70% of predicted, *n* (%)	65 (8)	15 (7)	0.78
ILD on HRCT, *n* (%)	305 (37)	71 (30)	0.33
PAH, *n* (%)	57 (7)	18 (8)	0.67
SSc-specific autoantibodies			
Anti RNA polymerase III, *n* (%)	62 (7)	29 (12)	0.23
Anti-centromere, *n* (%)	392 (47)	96 (41)	0.34
Anti-topoisomerase, *n* (%)	165 (20)	56 (24)	0.57
Treatment at baseline			
Calcium channel blockers, *n* (%)	305 (37)	95 (40)	0.67
H2 receptor blocker, *n* (%)	256 (31)	72 (31)	0.78
ET-1 inhibitors and prostacyclin analogue, *n* (%)	97 (12)	19 (5)	0.23
Proton pump inhibitor, *n* (%)	298 (36)	83 (35)	0.66
Methotrexate, *n* (%)	78 (9)	24 (10)	0.71
Mycophenolate mofetil, *n* (%)	38 (5)	13 (6)	0.81
Azathioprine, *n* (%)	13 (2)	2 (1)	0.91
Corticosteroids, *n* (%)	85 (10)	27 (11)	0.81
Cyclophosphamide, *n* (%)	11 (1)	10 (4)	0.54
Hydroxychloroquine, *n* (%)	27 (3)	7 (3)	0.68

*n* = 454 patients of the Leiden University Medical Center and *n* = 380 patients of the Oslo University Hospital; DLCO: single-breath diffusing lung capacity for carbon monoxide; ET-1: endothelin receptor, FVC: forced vital capacity; HRCT: high resolution computed tomography; ILD: interstitial lung disease; IQR: interquartile range; *n*: number; PAH: pulmonary arterial hypertension; SD: standard deviation; ± based on modified Rodnan Skin Score >15 points.

### GIT symptom severity among SSc patients

**Fig. 1:**
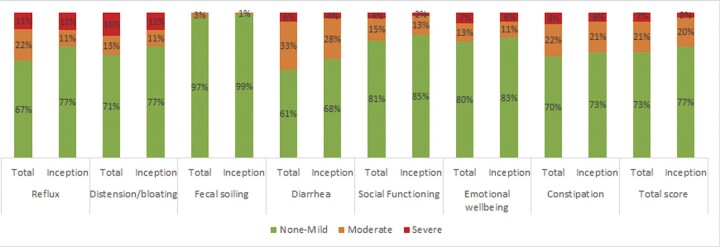
Severity of gastrointestinal involvement at baseline in the total cohort and the inception cohort Disease severity at baseline according to the UCLA GIT 2.0 score on every subdomain in the total and the inception cohort. This figure shows the percentage of SSc patients with non-mild, moderate and severe gastro-intestinal involvement for each subdomain at baseline.

We assessed all baseline values of all the sub-items of the UCLA GIT score both in the total cohort and inception cohort. At baseline, the total UCLA GIT score was equivalent to non-mild GIT symptoms in most of the patients (73%, *n* = 601), with 11% of these patients reporting a score of zero. Twenty-one percent of patients (*n* = 175) reported moderate GIT symptom burden, and 7% severe (*n* = 58). As shown in Fig. 1, the frequency of severe symptom burden varied considerably between the UCLA GIT subdomains. In the inception cohort, 77% (*n* = 181) reported non-mild GIT symptoms, 20% (*n* = 47) reported moderate GIT symptom burden and 3% severe symptom burden. The prevalence and distribution of GIT symptoms did not differ for the majority of GIT subdomains between the total cohort and the inception cohort. Among patients in the inception cohort, frequency of moderate-severe reflux and distension/bloating was lower (Fig. 1).

### Baseline disease characteristics associated with total GIT symptom burden

By multivariable analysis in the total cohort, we found that female sex [OR 1.76 (1.04–2.98)], ever smoking [OR 1.69 (1.19–2.41)], presence of ACA [OR 2.07 (1.34–3.19)] and corticosteroid use [OR 1.92 (1.18–3.12)] were significantly associated with moderate-severe total GIT symptom burden at baseline and CCB use [OR 0.55 (0.39–0.83)] seemed to be protective (Supplementary Table S2, available at *Rheumatology* online).

To control for the influence of longstanding disease, we conducted all analyses in the inception cohort. Here, we found that female sex [OR 8.5 (1.1–36.01)] and ever smoking [OR 2.9 (1.2–7.3)] were associated with GIT symptom severity (Table 2). Standard of care therapies were not associated with GIT symptom severity at baseline in the inception cohort. To assess whether any of the specific organ manifestations of SSc associate with GIT symptom severity, we applied multivariable logistic regression with each of the single organ manifestations. Presence of digital ulcers showed significant associations with total GIT symptom score [OR 1.5 (1.1–2.2)] and with distension/bloating [OR 1.7 (1.2–2.4)].

**Table 2:** Logistic regression of baseline characteristics associated with moderate/severe total UCLA GIT symptom score

	Univariable			Multivariable		
	Moderate/severe total GIT symptom score					
	OR	95% CI	Significance *P*-value	OR	95% CI	Significance *P*-value
Female	**3.03**	**1.22, 7.5**	0.01^*^	**8.5**	**1.1, 36.01**	<0.001^*^
Age, years	1.01	0.99, 1.03	0.30	—	—	—
Disease duration, yrs	0.61	0.37, 1.01	0.06	0.65	0.43, 1.11	0.13
Raynaud duration, yrs	1.01	0.97, 1.04	0.68	—	—	—
Smoking ever	**1.73**	**1.13, 3.68**	0.03^*^	**2.9**	**1.2, 7.2**	<0.001^*^
Diffuse subset^a^	0.89	0.56, 1.40	0.89	—	—	—
Disease severity^b^	1.22	0.63, 2.45	0.56	1.34	0.78, 2.66	0.62
Weight loss (>10% in 1 year)	0.67	0.25, 1.82	0.43	—	—	—
Haemoglobin level	0.70	0.41, 1.20	0.20	—	—	—
Myositis	1.01	0.32, 3.22	0.98	—	—	—
Anti-centromere antibody	1.86	0.98, 3.4	0.07	2.01	0.93, 5.32	0.11
Anti-topoisomerase antibody	1.52	0.71, 3.25	0.28	—	—	—
Anti-RNApIII antibody	0.54	0.24, 1.23	0.14	—	—	—
Proton pump inhibitor	0.65	0.25, 1.20	0.17	—	—	—
H2 receptor blocker	1.62	0.73, 3.56	0.23	—	—	—
ACE-inhibitor	0.89	0.41, 1.96	0.78	—	—	—
Calcium channel blocker	1.06	0.57, 1.98	0.84	—	—	—
Mycophenolate mofetil	0.38	0.09, 1.03	0.06	0.43	0.08, 1.05	0.09
Methotrexate	0.70	0.28, 1.78	0.45	—	—	—
Azathioprine	0.26	0.22, 1.03	0.09	0.43	0.34, 1.18	0.23
Corticosteroids	0.65	0.27, 1.57	0.33	—	—	—
Cyclophosphamide	1.09	0.22, 5.35	0.91	—	—	—
Hydroxychloroquine	0.79	0.15, 4.07	0.77	—	—	—
Prostacyclin and ET-1 inhibitor	0.34	0.09, 1.28	0.11	—	—	—

a Disease subset: entered as ordinal variable with order: non-cutaneous, limited and diffuse cutaneous subset. CI: confidence interval, OR: odds ratio.

b Disease severity is a compound variable which included: interstitial lung disease, pulmonary arterial hypertension, renal crisis, severe skin disease or presence of digital ulcers. Medication is entered as yes or no, with a yes if the medication was used in the same year as the GIT questionnaire was completed. ACE: angiotensin-converting-enzyme; ET-1: endotheline receptor; IQR: interquartile range; SD: standard deviation. In bold are the significant associations.

* remains significant after Holm–Bonferroni correction.

### Association of baseline disease characteristics and standard of care treatment with GIT symptom burden assessed in subdomains

Next, we evaluated associations of baseline clinical characteristics separately for each of the individual subdomains of the UCLA GIT questionnaire in the total cohort, as this cohort closely reflects daily clinical practice. Here, we corrected for disease duration and disease severity in our multivariate analyses (Supplementary Tables S2–S9, available at *Rheumatology* online). We identified dcSSc as a risk factor for more severe distension/bloating, diarrhoea and for social dysfunctioning. Presence of ACA was identified as a risk factor for severity in every subdomain except for constipation.

Regarding standard of care treatments, we found that ever use of corticosteroids associated with more severe faecal soilage [OR 3.91 (1.27–12.08)]. The use of CCB was protective against severe distention/bloating [OR 0.56 (0.37–0.83)] and diarrhoea [OR 0.61 (0.44–0.85)]. Regarding, reflux medication, we found that ever use of proton pump inhibitor associated with more severe reflux [OR 1.39 (1.07–1.80)] and with more severe faecal soilage symptoms [OR 3.07 (1.28–7.49)]. Ever use of H2 receptor blockers was also associated with more severe reflux [OR 1.71 (1.28–2.32)], but was protective for severe distension/bloating [OR 0.66 (0.47–0.93)]. The other therapeutics assessed were not associated with severity in any of the seven sub-items.

### Development of GIT symptoms over time

**Fig. 2:**
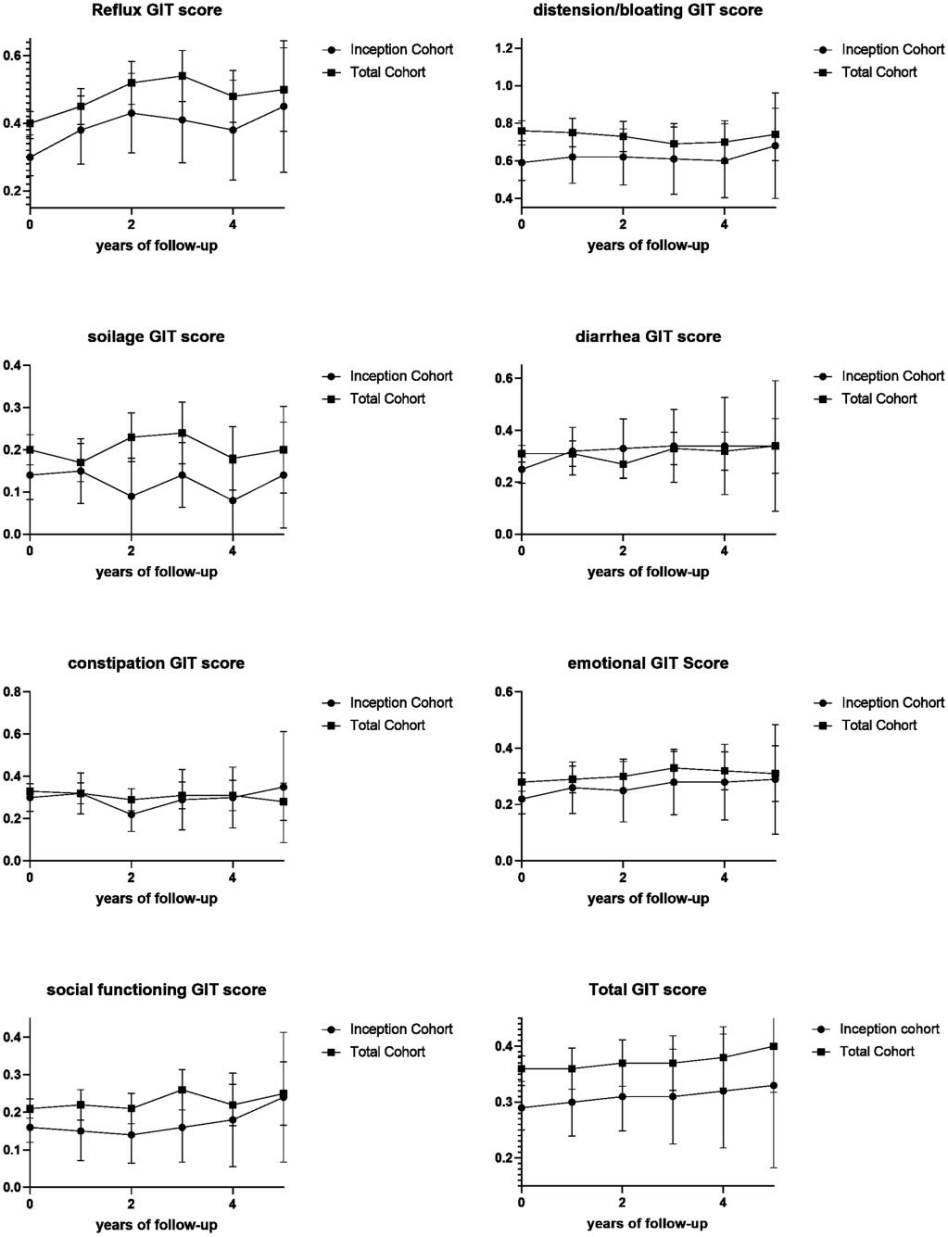
Mean scores per GIT subdomain over the follow-up period in the inception cohort and the total cohort Mean scores per GIT subdomain over the follow-up period in the inception and the total cohort. Higher scores indicate worse GIT symptoms, lower scores indicate lesser GIT symptoms.

In the total cohort, after one year of follow-up, 24% of patients had clinically important progression, defined by higher total GIT scores compared with baseline, with sub-item analyses showing progression of reflux symptoms in 25%, distension/bloating in 32% and constipation in 20%. To evaluate symptom burden evolution early in the disease course, we next evaluated progression of GIT symptoms in the inception cohort. In the inception cohort, after one year of follow-up, 27% of patients had clinically important GIT symptom progression, defined by increase in total GIT score from baseline, with sub-item analysis showing progression of reflux symptoms in 26%, distension/bloating in 29% and constipation in 21%. As shown by Fig. 2 the mean scores for the different subdomains vary greatly over time. All subdomains show higher scores after 5 years, indicating gradual worsening of GIT symptoms over time, and this is supported by the slope of the mean total score (Fig. 2). GIT symptom progression was not associated with GIT symptoms severity at baseline, neither for the total GIT score [OR 0.8 (0.3–2.6)], nor for any of the subdomains.

### Predictors for increasing GIT symptom burden over time

The multivariable linear mixed-effect model analyses in the total cohort showed that female gender and ACA positivity were predictors for worsening of GIT symptoms (Supplementary Table S10, available at *Rheumatology* online). None of the other variables were predictive for time-dependent progression in total GIT or subdomain scores.

The same analysis was performed in the inception cohort (Table 3). Here, of the variables of interest only treatment with CCB was identified as a predictor of marginal worsening of total GIT symptoms, with significant interaction effects between time and CCB treatment. The estimated difference of 0.04 in total GIT symptom score between CCB-treated and CCB-untreated patients was below the MCID previously defined for UCLA GIT [30].

**Table 3:** Linear mixed model in the inception cohort of systemic sclerosis patients

Predictor variable	Univariable			Multivariable		
	Coefficient	95% CI	*P*-value	Coefficient	95% CI	*P*-value
Sex	0.02	–0.03, 0.06	0.52			
Age	0.0003	–0.001, 0.002	0.71			
Disease duration	–0.011	–0.04, 0.01	0.37			
Ever smoking	0.04	–0.003, 0.08	0.07			
Anti-centromere antibody	–0.004	–0.04, 0.04	0.85			
Anti-topoisomerase antibody	0.006	–0.04, 0.05	0.81			
Skin involvement^a^	0.001	–0.0004, 0.003	0.13			
Diffuse disease subset	**0.04**	**–0.0008**, **0.09**	**0.05**	0.03	–0.001, 0.08	0.12
ESR	–0.0001	–0.0005, 0.0002	0.43			
Myositis	0.04	–0.05, 0.12	0.41			
PAH	0.05	–0.02, 0.13	0.18			
ILD	0.02	–0.02, 0.06	0.41			
ACE	0.02	–0.02, 0.06	0.33	0.02	–0.02, 0.06	0.29
CCB	**0.04**	**0.001**, **0.08**	**0.04**	**0.04**	**0.0006**, **0.08**	**0.04**
PPI	0.02	–0.02, 0.06	0.34	0.02	–0.02, 0.06	0.22
ET-1	0.05	–0.02, 0.13	0.22	0.05	–0.03, 0.14	0.19
H2 blocker	0.0003	–0.04, 0.04	0.99	0.002	–0.04, 0.04	0.94
Corticosteroids	0.02	–0.03, 0.06	0.48	0.02	–0.03, 0.06	0.46
Methotrexate	–0.011	–0.06, 0.04	0.68	0.006	–0.04, 0.05	0.79
Azathioprine	–0.06	–0.14, 0.05	0.18	–0.05	–0.13, 0.03	0.22
Hydroxychloroquine	–0.03	–0.10, 0.03	0.28	–0.04	–0.10, 0.03	0.26
MMF	–0.01	–0.08, 0.06	0.76	–0.006	–0.07, 0.06	0.87
Cyclophosphamide	0.04	–0.006, 0.09	0.090	0.04	–0.006, 0.09	0.08

Coefficients shown are the interactions terms (predictor × time). Mean between group change during three years of follow-up. Separate multivariable regression analyses with adjustment for time, age, and sex. ACE: angiotensin-converting-enzyme inhibitors; CCB: calcium channel blockers; ET-1: endotheline receptor antagonist; ILD: interstitial lung disease; mRSS: modified Rodnan Skin Score; PAH: pulmonary arterial hypertension; PPI: proton pump inhibitors. Time effects of the mixed model can be found in supplementary file.

a Assessed by the modified Rodnan skin score. Significant results are not significant after Bonferroni–Holm correction. Bold indicates significance *P* value M 0.05.

### Impact of immunomodulatory treatment on development of GIT symptom burden over time

In the total cohort, 83% (*n* = 692) were treatment-naïve at baseline. Over the follow-up period, the frequency of unexposed patients was reduced to 68% (*n* = 567). In the inception cohort, 81% (*n* = 192) were treatment-naïve at baseline and 48% (*n* = 72) remained so at follow-up. We first evaluated changes in UCLA GIT scores from baseline to follow-up in patients who were naïve for immunomodulatory treatment across the observation period. We found that more patients at each subsequent visit had worsened since baseline over the first three years in patients naïve for immunomodulatory treatment, except for the diarrhoea and emotional subdomain (Supplementary Figure S1, available at *Rheumatology* online).

To evaluate impact of initiation with immunomodulatory therapies, we determined GIT symptom progression in treatment-exposed patients. Typical indications for immunomodulatory therapies in SSc are severe skin and lung disease. Hence, it was not unexpected to find that patients exposed to treatment were more often dcSSc and positive for ATA and had more frequently ILD than the treatment-naïve patients, while age and sex were comparable.

**Fig. 3:**
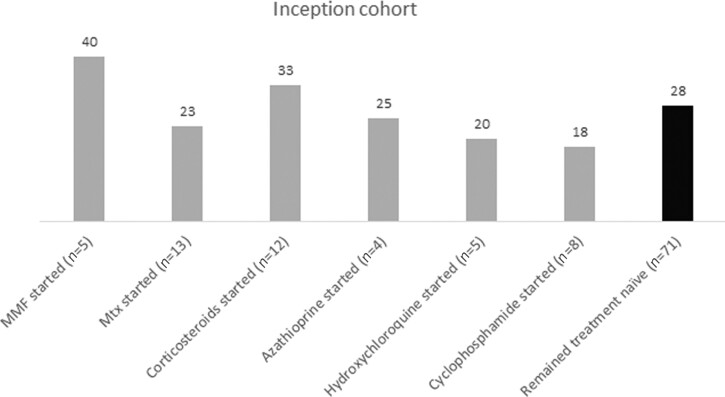
Progressors (inception) for the total GIT score after 1 year stratified for immunomodulatory treatment Percentage of progressors in inception cohort. Inception patients with available UCLA GIT after one year (*n* = 118); *n* = 71 remained treatment-naïve, *n* = 47 started with immune-modulating therapy. No treatment are those patients who remained treatment-naïve.

In both the total cohort and the inception cohort we found no significant difference in GIT symptom progression between immunomodulatory treatment-naïve patients and patients exposed to methotrexate, MMF, azathioprine, hydroxychloroquine, corticosteroids or cyclophosphamide after one year of follow-up. Numerically, more patients that started with corticosteroids or MMF had progressive GIT symptoms after one year in the inception cohort (Fig. 3).

## Discussion

GIT involvement is reported as highly frequent in SSc, with major impact on patient’s quality of life and survival, but so far, no studies have assessed the exact burden of GIT disease among SSc patients, disease characteristics impacting GIT symptoms, the disease course with predictors for increasing GIT symptom burden and the impact of immunomodulatory treatment on development of GIT burden over time in a large multicentre study including a large amount of newly diagnosed patients.

Here, we show that about one-third of patients in a large, unselected SSc population report high GIT symptom burden early in the disease course, with reflux and distention/bloating as the most troublesome early symptoms. To answer which disease characteristics impact GIT symptom burden in SSc, we performed the first ever systematic assessment of associations between SSc characteristics including standard-of-care therapies and GIT symptoms in two large SSc cohorts mapping the whole SSc population. Interestingly, we found that corticosteroid use might associate with high GIT symptom burden. The association with corticosteroids was not identified in the inception cohort subset. The smaller sample size could be one explanation, another could be that GIT side effects of corticosteroids accumulate over time. A recent cross-sectional study in SSc did observe increasing GIT symptom burden among ACA positive patients [32]. In their analyses, no correction for disease duration or other confounders was applied, while mean disease duration at baseline in this cohort was 12 years. We confirm in our total cohort that GIT symptom burden is worse with ACA presence. However, in the inception cohort this association was not statistically significant. This might be explained by a lack of power, given the large confidence interval. However, we hypothesize that, given the observation that GIT symptoms increase over time with growing disease duration, this association might only become clear with longer disease duration. We can only speculate about the background of this association: (i) ACA-positive SSc patients might have a longer ‘prodrome’ as a consequence of a more indolent disease course. Indeed, disease duration since onset of Raynaud’s phenomenon was significantly longer in ACA positive patients compared with ATA positive patients. On the other hand, the association between ACA and GIT symptoms remained significant after correcting for disease duration. (ii) ACA are directly implicated in SSc-related vasculopathy and consequently GIT symptom severity (still unknown) (33–39).

Next, we prospectively mapped GIT symptom behaviour over time including both our total SSc cohort mirroring the daily clinical patient cohort and the inception cohort. Over one year observation, 27% of patients reported worsening of GIT symptoms. Interestingly, this worsening occurred independent of baseline GIT symptom severity and disease duration, which shows that although GIT complications are reported to increase with disease duration, patients experience important burden already early in the disease course. Despite including many clinical characteristics, identifying predictors for worsening of GIT symptoms was challenging. We identified female sex, ACA, smoking and CCB use as predictors for worsening of GIT symptoms. There were no differences in GIT symptom progression between patients who were treatment-naïve at all time-points and patients who started immunomodulatory treatment during follow-up. These results argue that immunomodulatory treatment for SSc does not seem to have a major impact on GIT symptom evolution in SSc. The exact aetiology of GIT symptoms in SSc is and remains largely unknown and therefore can be influenced by multiple (unknown) factors. Previous data on this subject are highly limited, but it appears that our results are in line with McMahan *et al.* who showed no effect of immunomodulatory treatment on severe dysmotility assessed by the Medsger activity score (37). Notably, that study did not use a patient-reported GIT outcome measure, it did not include treatment-naïve patients, and it did not assess effects of vasodilatory treatments.

The identification of a high symptomatic burden from early in the disease course and, even more importantly, the progressive behaviour of the GIT symptoms both in early and in longstanding disease of our study has impact at several levels. Firstly, it is of major clinical relevance for physicians following these patients to actively assess a patient’s symptoms both at baseline regardless disease duration and over the disease course to individually tailor their management. Secondly, our results argue for the necessity of multidisciplinary team (MDT) assessment including rheumatologists and gastroenterologists. MDT assessments are important and well-functioning for other diseases, like Crohn’s disease and in ILD [40, 41]. We strongly advocate to build up MDT for GIT involvement in all SSc expert centres, and initiate work on recommendations for its management, as also highlighted in the recently published international standard for longitudinal follow-up of SSc patients [42]. Lastly, knowledge of the natural course of GIT symptoms is a prerequisite to identify patients for study inclusion and to determine outcome of clinical trials assessing novel treatment options for GIT symptoms in SSc. This is particularly important in times of evolving new therapeutic options, like faecal microbiota transplantation [43, 44].

The mechanisms behind the GIT involvement in SSc are not well understood, but appear multifactorial. Our study does not add pathomechanistical insights but opens some interesting hypotheses. Vasculopathy is an important factor in SSc pathogenesis, and some reports indicate associations between GIT symptoms and progressive vasculopathy [32, 33]. Interestingly, in our study, patients showing progression of GIT symptoms also had digital ulcers more often, while no association was found with other organ manifestations such as ILD or skin involvement. Although this is not a mechanistic study, we speculate that the digital ulcer association, and possibly also the effects of CCB in the cross-sectional data set, implicates vasculopathy in the pathophysiology of SSc GIT disease.

Our study is not without limitations. The UCLA GIT is a validated questionnaire; however, it remains a self-reported questionnaire and this can always introduce bias. The UCLA GIT captures symptoms in the past seven days; we included annual follow-up which might not capture all short-term changes. Many assessments were performed in this study; by using Bonferroni correction we have reduced the risk of type I errors. A relatively low percentage of patients experienced faecal soilage, which might be underestimated due to recruitment bias and patients’ reluctance to talk about this symptom. Using the MCID could still miss patients with clinically relevant GIT development, as there is an inherent uncertainty around MCID estimates [30]. Unfortunately, objective investigations are not routinely performed in these cohorts and therefore we were not able to evaluate clinical associations with objective measurements. In SSc GIT disease, there is a lack of clinical associations with objective measurements and we suggest that for a better understanding of the aetiology, this would be a next step to evaluate and this highlights a clear clinical unmet need for further studies to clarify this. We should also be aware of the possibility of confounding by indication in the analyses evaluating GIT treatment and GIT symptom progression as patients with more severe reflux symptoms are also more likely to receive GIT treatment. Finally, the medication included in this study was based on standard of care therapy in SSc and availability in the database. Unfortunately, GIT medication outside of gastroesophageal reflux disease medication has only been collected since 2018 in the Leiden cohort, including ever use of metoclopramide, domperidone and antibiotics of 483 SSc patients, 4% used domperidone (*n* = 20), 4% used metoclopramide (*n* = 16), 8% laxatives (*n* = 42), loperamide 1% (*n* = 4) and 1% used antibiotics for bacterial overgrowth (*n* = 4). Given these low frequencies, no major impact is expected.

Our study also has major strengths, including the large and prospective cohort, and the amount of questionnaires at baseline and over the observation period with very few missing data (<5%) and a high compliance rate (>90%). Biomarkers for GIT disease activity are still not defined, making it challenging to assess the effects of existing therapies. The UCLA GIT questionnaire allows for a standardized assessment of important clinical response measures in SSc and may play a role in informing both clinical practice and trial design.

In conclusion, our data provide important insights regarding the high frequency of severe GIT symptoms early in the disease course, and the progressive nature of GIT symptoms in patients with SSc. We confirm ACA-positive patients, female patients and/or smoking patients are specifically at risk for GIT symptoms, but very few other variables can help identify patients at risk of disease progression. We strongly advocate to build up MDT for GIT in all SSc expert centres, and initiate work on recommendations for the management of this devastating organ affliction in SSc.

## Supplementary Material

keac118_Supplementary_Data

## Data Availability

Data are available upon reasonable request. All data relevant to the study are included in the article or uploaded as supplementary information.
