# Supplementary material for: Gastrointestinal symptom severity and progression in systemic sclerosis
Source: Rheumatology (Oxford). 2022 Mar 3;61(10):4024–34. doi: 10.1093/rheumatology/keac118 (PMC9789747; doi:10.1093/rheumatology/keac118)
Supplement: keac118_Supplementary_Data [file keac118_supplementary_data.docx]

**Supplementary file**

**Gastro-intestinal symptom severity and progression in systemic sclerosis**

**Data S1. UCLA GIT 2.0**

Clinical GIT involvement by the UCLA GIT score was defined as apparent if the patients reported symptoms resulting in a total score or at least one of the seven GIT items ≥0.01 and was segregated into none-mild (including patients with a score of 0, or <0.5 or for fecal incontinence and distention/bloating <1.01), moderate (≥0.5 or for fecal incontinence and distention/bloating ≥1.01) or severe GIT symptoms (> 1.01 or for distension/bloating > 1.61 or for fecal soiling > 2.01) (1).

The minimal clinical important difference (MCID) somewhat worse threshold was used to assess disease progression and cut-offs were defined as: Reflux: 0.19, Distension/bloating: 0.12, Diarrhoea: 0.07, Constipation: 0.13, Fecal Soilage 0.06, Emotional well-being: 0.16, Social functioning: 0.21, Total GIT score 0.12 (2).

**Data S2. Candidate variables**

The following variables were included as candidate baseline variables for associations with GIT symptoms and prediction of GIT symptom progression: sex, age at registration, disease duration since disease onset, smoking, SSc specific antibodies (anti-centromere antibody [ACA], anti-topoisomerase antibody [ATA], anti-RNA polymerase III antibody [ARA]), SSc subset (diffuse cutaneous and limited cutaneous SSc), myositis (defined as increased creatine kinase AND proximal muscle weakness and/or confirmed with muscle biopsy), weight loss (>10% of body weight in 1 year), and hemoglobin level (3-10).

**Data S3. Organ involvement screening**

Skin involvement was assessed using the modified Rodnan skin score (mRSS), a validated measure of skin thickening in SSc.(11) Skeletal myopathy was considered present when patients had an abnormal creatinine phosphokinase (CPK) and muscle weakness and/or abnormal electromyography or muscle biopsy. The presence of interstitial lung disease (ILD) was considered present if a high resolution CT scan of the thorax showed ground glass opacifications, honey combing and/or reticular infiltrations interpreted by an experienced radiologist. Pulmonary function tests were performed by every patient, and the percent predicted FVC and DLCO were determined. Pulmonary arterial hypertension was defined as an increase in mean pulmonary arterial pressure (mPAP) ≥ 25 mmHg at rest as assessed by RHC; including presence of pre-capillary PH, defined by a pulmonary capillary wedge pressure (PCWP) ≤15 mmHg and a PVR >3 Wood units (WU) on RHC*.*

**Data S4. Generating univariable and multivariable model**

At the first stage, we used subject‐specific knowledge to derive a list of independent variables which in principle are relevant as predictors or adjustment variables for the study in question. This list was also based on the availability of variables. With three researchers (NvL, JVB, AMH) we went through the list and critically questioned the role and further properties of each of the variables, such as chronology of measurement collection, quality of measurement, or availability also to the “user” of the model. Not for all independent variables pre-clinical knowledge was available, therefore we carried out an univariable analysis and selected those that reached a certain level of significance ( p <0.10). We selected the priori covariates based on biological mechanism, clinical expertise, evidence from published articles, and the univariable model. We checked the assumptions of linearity in logit for each continuous covariate and we checked for interactions. The proportional odds assumption was met. The following covariates were fixed in the model based on their clinical relevance and expert opinion: age, sex, disease duration NR, cutaneous subtype, smoking, autoantibodies and disease severity. The medication covariates were included based on hypothesis testing, frequency of use in clinical practice and availability.

| **Table S1:** Baseline demographic and clinical characteristics of the total systemic sclerosis cohort, stratified by origin (Leiden University Medical Center, LUMC, or Oslo University Hospital, OUH) | | | |  |
| --- | --- | --- | --- | --- |
|  | **LUMC** | **OUH** | | **P** |
| **Demographic** | n=454 | n=380 | |  |
| Female, n (%) | 336 (81) | 307 (81) | | 0.9 |
| Age, yrs mean (SD) | 55 (14) | 56 (13) | | 0.8 |
| SSc disease duration at inclusion, median (IQR) | 3.8 (1.1-10.2) | | 4.3 (1.8-8.8) | 0.6 |
| Smoking, ever n (%) | 245 (55) | 122 (45) | | 0.4 |
| **Organ involvement** |  |  | |  |
| Diffuse cutaneous SSc, n (%) | 88 (21) | 84 (22) | | 0.5 |
| Severe skin involvement, n (%)± | 37 (8) | 59 (16) | | 0.2 |
| Myositis, n (%) | 13 (3) | 48 (13) | | 0.2 |
| DLCO% < 60% of predicted, n (%) | 134 (32) | 100 (29) | | 0.6 |
| FVC% < 70% of predicted, n (%) | 36 (9) | 26 (8) | | 0.8 |
| ILD on HRCT, n (%) | 180 (39) | 120 (32) | | 0.5 |
| PAH, n (%) | 20 (4) | 34 (9) | | 0.6 |
| **SSc specific autoantibodies** |  |  | |  |
| Anti RNA polymerase III, n (%) | 25 (6) | 37 (10) | | 0.7 |
| Anti-centromere, n (%) | 184 (40) | 208 (55) | | 0.3 |
| Anti-topoisomerase, n (%) | 117 (26) | 48 (13) | | 0.3 |
| **Treatment at baseline** |  |  | |  |
| Calcium Channel Blockers, n (%) | 190 (42) | 114 (30) | | 0.4 |
| H2 receptor blocker, n (%) | 150 (33) | 106 (28) | | 0.5 |
| ET-1 inhibitors&prostacyclin analogue, n (%) | 62 (14) | 56 (15) | | 0.7 |
| Proton Pump Inhibitor, n (%) | 179 (39) | 121 (32) | | 0.6 |
| Methotrexate, n (%) | 79 (17) | 20 (5) | | 0.2 |
| Mycophenolate mofetil, n (%) | 28 (6) | 19 (5) | | 0.5 |
| Azathioprine, n (%) | 14 (3) | 5 (1) | | 0.7 |
| Corticosteroids, n (%) | 71 (16) | 37 (10) | | 0.6 |
| Cyclophosphamide, n (%) | 20 (4) | 6 (2) | | 0.8 |
| Hydroxychloroquine, n (%) | 32 (7) | 3 (1) | | 0.7 |
| DLCO= single-breath diffusing lung capacity for carbon monoxide, ET-1=endothelin receptor, FVC= forced vital capacity, ILD=interstitial lung disease, HRCT= high resolution computed tomography, PAH= pulmonary arterial hypertension, n=number, SD=standard deviation; IQR= interquartile range. ± based on modified Rodnan Skin Score > 15 points. | | | |  |

| **Table S2:** Logistic regression analyses of baseline characteristics associated with moderate/severe total UCLA GIT symptom score in the 834 systemic sclerosis patients (total cohort) from the Leiden and Oslo cohort | | | | | | |
| --- | --- | --- | --- | --- | --- | --- |
|  |  | Univariable |  |  | Multivariable |  |
|  | **Moderate / severe total GIT symptom score** | | | | | |
|  | OR | 95% CI | Significance  p-value | OR | 95% CI | Significance  p-value |
| Female | **1.97** | **1.25-3.10** | **<0.001*** | **1.76** | **1.04-2.98** | **0.03** |
| Age, Years | 0.99 | 0.99-1.004 | 0.28 | 0.99 | 0.98-1.01 | 0.07 |
| Disease duration, yrs | **1.04** | **1.02-1.06** | **<0.001*** | **1.05** | **1.03-1.07** | **<0.001*** |
| Raynaud duration, yrs | **1.01** | **1.00-1.02** | **0.04** | **-** | **-** | **-** |
| Smoking ever | **1.50** | **1.07-2.08** | **0.02** | **1.69** | **1.19-2.41** | **<0.001*** |
| Diffuse subset* | 1.11 | 0.66-1.87 | 0.69 | 1.88 | 0.94-3.76 | 0.08 |
| Disease severity# | 1.13 | 0.82-1.54 | 0.46 | 1.02 | 0.70-1.48 | 0.91 |
| Weight loss (>10% in 1 year) | 1.20 | 0.74-1.97 | 0.46 | - | - | - |
| Hemoglobine level | 1.10 | 0.88-1.39 | 0.40 | - | - | - |
| Myositis | 1.12 | 0.64-1.96 | 0.69 | - | - | - |
| Anti-centromere antibody | **1.77** | **1.30-2.39** | **<0.001*** | **2.07** | **1.34-3.19** | **<0.001*** |
| Anti-topoisomerase antibody | **0.57** | **0.37-0.86** | **0.01** | 0.78 | 0.46-1.33 | 0.36 |
| Anti-RNApIII antibody | 0.90 | 0.41-1.99 | 0.79 | - | - | - |
| Proton pump inhibitor | 1.26 | 0.95-1.67 | 0.11 | - | - | - |
| H2 receptor blocker | 0.92 | 0.68-1.24 | 0.58 | - | - | - |
| ACE-inhibitor | 0.75 | 0.53-1.06 | 0.10 | - | - | - |
| Calcium channel blocker | 0.77 | 0.56-1.07 | 0.10 | **0.56** | **0.39-0.83** | **<0.001*** |
| Mycophenolate mofetil | 1.23 | 0.64-2.33 | 0.54 | - | - | - |
| Methotrexate | 0.88 | 0.55-1.42 | 0.60 | - | - | - |
| Azathioprine | 0.53 | 0.15-1.86 | 0.32 | - | - | - |
| Corticosteroids | 1.25 | 0.83-1.88 | 0.10 | **1.92** | **1.18-3.12** | **<0.001*** |
| Cyclophosphamide | 0.77 | 0.31-1.93 | 0.58 | - | - | - |
| Hydroxychloroquine | 0.99 | 0.48-2.09 | 0.99 | - | - | - |
| Prostacyclin & ET-1 inhibitor | 1.57 | 0.92-2.67 | 0.09 | 1.49 | 0.96-2.33 | 0.13 |
| *Disease subset: entered as ordinal variable with order: non-cutaneous, limited and diffuse cutaneous subset. CI=confidence interval, OR= odds ratio. # disease severity is a compound variable which included: interstitial lung disease, pulmonary arterial hypertension, renal crisis, severe skin disease or presence of digital ulcers. Medication is entered as yes or no, with a yes if the medication was used in the same year as the GIT questionnaire was completed. ACE= angiotensin-converting-enzyme, ET-1=endotheline receptor. IQR= interquartile range, SD=standard deviation. In bold are the significant associations. * remains significant after Holm-Bonferroni correction. | | | | | | |

| **Table S3.**  Logistic regression analyses of baseline characteristics associated with moderate or severe reflux symptom score in the 834 systemic sclerosis patients from the Leiden and Oslo cohort | | | | | | |
| --- | --- | --- | --- | --- | --- | --- |
|  | Univariable | | | Multivariable | | |
|  | **Moderate or severe reflux symptom score** | | | | | |
|  | **OR** |  | **Significance** | **OR** |  | **Significance** |
|  |  | **95% CI for OR** |  |  | **95% CI for OR** |  |
| Female | **1.75** | **1.16-2.64** | **0.01** | **1.60** | **1.00-2.55** | **0.05** |
| Age, Years | 0.99 | 0.99-1.003 | 0.72 | 0.99 | 0.98-1.01 | 0.46 |
| Disease duration, years | **1.02** | **1.007-1.040** | **0.01** | **1.02** | **1.004-1.04** | **0.02** |
| Raynaud duration, years | **1.01** | **1.005-1.034** | **0.03** |  |  |  |
| Smoking ever | 1.17 | 0.87-1.58 | 0.31 | 1.19 | 0.86-1.63 | 0.30 |
| Disease subset* | 1.04 | 0.64-1.68 | 0.89 | 1.43 | 0.78-2.62 | 0.25 |
| Disease severity# | 1.15 | 0.86-1.55 | 0.35 | 1.09 | 0.78-1.54 | 0.62 |
| Weight loss (>10% in 1 year) | **1.74** | **1.09-2.77** | **0.02** | **2.20** | **1.33-3.63** | **<0.001*** |
| Hemoglobin levels | 0.89 | 0.72-1.12 | 0.32 | - | - | - |
| Myositis yes/no | 0.63 | 0.13-3.07 | 0.60 | - | - | - |
| Anti-centromere antibody | **1.71** | **1.22-2.27** | **<0.001*** | **1.76** | **1.10-2.27** | **0.01** |
| Anti-topoisomerase antibody | **0.51** | **0.34-0.76** | **<0.001*** | **0.57** | **0.34-0.90** | **0.02** |
| Anti-RNApIII | 0.72 | 0.41-1.29 | 0.28 | - | - | - |
| Proton pump inhibitor | **1.39** | **1.07-1.80** | **0.01** | 1.16 | 0.82-1.66 | 0.40 |
| H2 receptor blocker | **1.71** | **1.28-2.32** | **<0.001*** | **1.71** | **1.23-2.59** | **0.003*** |
| ACE inhibitor | 1.17 | 0.82-1.63 | 0.38 | - | - | - |
| Calcium channel blocker | 1.09 | 0.84-1.41 | 0.54 | - | - | - |
| Immunosuppressiva combined | 1.34 | 0.93-1.93 | 0.12 | - | - | - |
| Prostacyclin & ET-1 inhibitor | 1.47 | 0.89-2.42 | 0.22 | - | - | - |
| Univariable and multivariable logistic regression. *Disease subset: entered as ordinal variable with order: non-cutaneous, limited and diffuse subset. # disease severity is a compound variable which included: ILD with FVC<70%, PAH, renal crisis, mRSS>20 or presence of digital ulcers. Medication are entered as yes or no, with a yes if the medication was used in the same year as the GIT questionnaire was completed. * significant after Holm-Bonferroni correction. | | | | | | |

| **Table S4.**  Logistic regression analyses of baseline characteristics associated with moderate or severe distension/bloating symptom score in the 834 systemic sclerosis patients from the Leiden and Oslo cohort | | | | | | |
| --- | --- | --- | --- | --- | --- | --- |
|  | Univariable | | | Multivariable | | |
|  | **Moderate or severe distention/bloating symptom score** | | | | | |
|  | **OR** |  | **Significance** | **OR** |  | **Significance** |
|  |  | **95% CI for OR** |  |  | **95% CI for**  **OR** |  |
| Female | **2.34** | **1.48-3.67** | **<0.001*** | **2.38** | **1.40-4.03** | **<0.001*** |
| Age, Years | 0.99 | 0.99-1.003 | 0.14 | 0.99 | 0.98-1.00 | 0.06 |
| Disease duration, years | **1.02** | **1.002-1.04** | **0.03** | **1.03** | **1.01-1.05** | **<0.001*** |
| Raynaud duration, years | **1.00** | **1.00-1.02** | **0.04** |  |  |  |
| Smoking ever | **1.46** | **1.05-2.01** | **0.02** | **1.86** | **1.32-2.64** | **<0.001*** |
| Disease subset* | 1.25 | 0.75-2.08 | 0.38 | **2.29** | **1.16-4.53** | **0.02** |
| Disease severity | 1.31 | 0.97-1.78 | 0.08 | 1.26 | 0.88-1.82 | 0.21 |
| Weight loss (>10% in 1 year) | 0.93 | 0.54-1.61 | 0.80 | - | - | - |
| Hemoglobin | 0.86 | 0.70-1.06 | 0.17 | - | - | - |
| Myositis yes/no | 1.21 | 0.73-2.02 | 0.47 | - | - | - |
| ACA | **1.54** | **1.20-1.97** | **<0.001*** | **1.65** | **1.08-2.52** | **0.02** |
| ATA | 0.64 | 0.47-0.88 | 0.06 | 0.84 | 0.50-1.40 | 0.50 |
| Anti-RNApIII | 1.48 | 0.88-2.47 | 0.14 | - | - | - |
| Proton pump inhibitor | 1.10 | 0.85-1.43 | 0.45 | - | - | - |
| H2 receptor blocker | **0.66** | **0.47-0.93** | **0.02** | 0.85 | 0.56-1.27 | 0.42 |
| ACE inhibitor | 0.58 | 0.38-0.88 | 0.01 | 0.80 | 0.50-1.28 | 0.35 |
| Calcium channel blocker | **0.66** | **0.48-0.91** | **0.01** | **0.56** | **0.37-0.83** | **<0.001*** |
| Immunosuppressiva combined | 0.96 | 0.64-1.41 | 0.82 | - | - | - |
| Prostacyclin & ET-1 inhibitor | 1.30 | 0.83-2.09 | 0.29 | - | - | - |
| *Disease subset: entered as ordinal variable with order: non-cutaneous, limited and diffuse subset. # disease severity is a compound variable which included: ILD with FVC<70%, PAH, renal crisis, mRSS>20 or presence of digital ulcers. Medications are entered as yes or no, with a yes if the medication was used in the same year as the GIT questionnaire was completed. * Significant after Holm-Bonferroni correction. | | | | | | |

| **Table S5.**  Logistic regression analyses of baseline characteristics associated with moderate or severe constipation symptom score in the 834 systemic sclerosis patients from the Leiden and Oslo cohort | | | | | | | | | | |
| --- | --- | --- | --- | --- | --- | --- | --- | --- | --- | --- |
|  | Univariable | | | | Multivariable | | | | | |
|  | **Moderate or severe constipation symptom score** | | | | | | | | | |
|  | **OR** | **95% CI for OR** | **Significance P** | | **OR** | | **95% CI for OR** | **Significance**  **P** | | |
| Female | 1.76 | 1.16-2.67 | 0.008 | 1.51 | | 0.94-2.42 | | | 0.08 |  |
| Age, Years | 1.001 | 0.99-1.01 | 0.48 | 1.00 | | 0.99-1.01 | | | 0.97 |  |
| Disease duration, years | 1.01 | 0.99-1.03 | 0.29 | 1.02 | | 0.99-1.03 | | | 0.13 |  |
| Raynaud duration, years | 0.99 | 0.98-1.02 | 0.61 |  | |  | | |  |  |
| Smoking ever | 0.76 | 0.56-1.04 | 0.09 | 0.96 | | 0.67-1.30 | | | 0.68 |  |
| Disease subset* | 1.26 | 0.78-2.02 | 0.34 | 1.29 | | 0.65-2.56 | | | 0.47 |  |
| Disease severity | 1.30 | 0.96-1.76 | 0.09 | 1.21 | | 0.85-1.73 | | | 0.29 |  |
| Weight loss (>10% in 1 year) | 1.26 | 0.76-2.10 | 0.37 | - | | - | | | - |  |
| Hemoglobin | 0.86 | 0.68-1.09 | 0.21 | - | | - | | | - |  |
| Myositis yes/no | 1.11 | 0.62-1.99 | 0.73 | - | | - | | | - |  |
| ACA | 1.18 | 0.88-1.57 | 0.28 | 1.26 | | 0.84-1.89 | | | 0.27 |  |
| ATA | 0.67 | 0.45-0.99 | 0.05 | 1.04 | | 0.64-1.67 | | | 0.89 |  |
| Anti-RNApIII | **2.49** | **1.52-4.06** | **<0.001*** | **2.43** | | **1.31-4.50** | | | **0.01** |  |
| Proton pump inhibitor | 0.98 | 0.75-1.29 | 0.91 | - | | - | | | - |  |
| H2 receptor blocker | 0.76 | 0.48-1.08 | 0.11 | - | | - | | | - |  |
| ACE inhibitor | 0.72 | 0.51-1.02 | 0.25 | - | | - | | | - |  |
| Calcium channel blocker | 0.75 | 0.55-1.03 | 0.07 | 0.74 | | 0.52-1.06 | | | 0.09 |  |
| Immunosuppressiva combined | **0.60** | **0.40-0.92** | **0.02** | 0.76 | | 0.45-1.09 | | | 0.09 |  |
| Prostacyclin & ET-1 inhibitor | 1.34 | 0.73-2.23 | 0.58 | - | | - | | | - |  |
| *Disease subset: entered as ordinal variable with order: non-cutaneous, limited and diffuse subset. # disease severity is a compound variable which included: ILD with FVC<70%, PAH, renal crisis, mRSS>20 or presence of digital ulcers. Medications are entered as yes or no, with a yes if the medication was used in the same year as the GIT questionnaire was completed. * Significant after Holm-Bonferroni correction. | | | | | | | | | | |

| **Table S6.**  Logistic regression analyses of baseline characteristics associated with moderate or severe diarrhea symptom score in the 834 systemic sclerosis patients from the Leiden and Oslo cohort | | | | | | |
| --- | --- | --- | --- | --- | --- | --- |
|  | Univariable | | | Multivariable | | |
|  | **Moderate or severe diarrhea symptom score** | | | | | |
|  | **OR** | **95% CI for OR** | **Significance P** | **OR** | **95% CI for OR** | **Significance P** |
| Female | 1.06 | 0.74-1.53 | 0.74 | 1.05 | 0.70-1.60 | 0.80 |
| Age, Years | 0.99 | 0.98-1.004 | 0.21 | 0.99 | 0.98-1.01 | 0.13 |
| Disease duration, years | **1.01** | **1.01-1.04** | **0.01** | 1.02 | 1.01-1.04 | 0.05 |
| Raynaud duration, years | **1.02** | **1.01-1.06** | **0.02** |  |  |  |
| Smoking ever | 1.35 | 1.01-1.81 | 0.05 | 1.43 | 1.05-1.94 | 0.02 |
| Disease subset* | **2.27** | **1.37-3.76** | **<0.001*** | **2.59** | **1.40-4.79** | **<0.001*** |
| Disease severity | **1.39** | **1.05-1.85** | **0.02** | 1.33 | 0.96-1.85 | 0.08 |
| Weight loss (>10% in 1 year) | 1.40 | 0.87-2.26 | 0.16 | - | - | - |
| Hemoglobin | 1.21 | 0.95-1.53 | 0.12 | - | - | - |
| Myositis yes/no | 1.72 | 1.01-2.95 | 0.05 | 1.52 | 0.82-2.84 | 0.18 |
| ACA | 1.27 | 0.96-1.67 | 0.09 | **1.63** | **1.12-2.36** | **0.01** |
| ATA | 0.76 | 0.53-1.08 | 0.12 | 0.79 | 0.52-1.22 | 0.29 |
| Anti-RNApIII | 0.92 | 0.54-1.56 | 0.76 | - | - | - |
| Proton pump inhibitor | 0.97 | 0.73-1.30 | 0.84 | - | - | - |
| H2 receptor blocker | 0.80 | 0.59-1.10 | 0.17 | - | - | - |
| ACE inhibitor | 0.82 | 0.57-1.17 | 0.26 | - | - | - |
| Calcium channel blocker | 0.69 | 0.52-0.93 | 0.01 | **0.61** | **0.44-0.85** | **<0.001*** |
| Immunosuppressiva combined | 0.71 | 0.49-1.03 | 0.07 | 0.73 | 0.52-1.13 | 0.11 |
| Prostacyclin & ET-1 inhibitor | 1.03 | 0.61-1.70 | 0.87 | - | - | - |
| *Disease subset: entered as ordinal variable with order: non-cutaneous, limited and diffuse subset. # disease severity is a compound variable which included: ILD with FVC<70%, PAH, renal crisis, mRSS>20 or presence of digital ulcers. Medications are entered as yes or no, with a yes if the medication was used in the same year as the GIT questionnaire was completed. * Significant after Holm-Bonferroni correction. | | | | | | |

| **Table S7.**  Logistic regression analyses of baseline characteristics associated with moderate or severe fecal soilage symptom score in the 834 systemic sclerosis patients from the Leiden and Oslo cohort | | | | | | |
| --- | --- | --- | --- | --- | --- | --- |
|  | Univariable | | | Multivariable | | |
|  | **Moderate or severe fecal soilage symptom score** | | | | | |
|  | **OR** | **95% CI for OR** | **Significance P** | **OR** | **95% CI for OR** | **Significance P** |
| Female | 2.08 | 0.48-9.01 | 0.33 | 1.39 | 0.28-6.89 | 0.69 |
| Age, Years | **1.04** | **1.001-1.08** | **0.02** | 1.04 | 0.99-1.08 | 0.13 |
| Disease duration, years | **1.06** | **1.02-1.10** | **<0.001*** | **1.08** | **1.04-1.13** | **<0.001*** |
| Raynaud duration, years | **1.04** | **1.03-1.06** | **0.02** |  |  |  |
| Smoking ever | 1.69 | 0.66-4.33 | 0.28 | 1.44 | 0.52-3.99 | 0.48 |
| Disease subset* | 2.55 | 0.28-23.08 | 0.41 | 13.36 | 0.89-201.61 | 0.06 |
| Disease severity | 0.92 | 0.37-2.32 | 0.89 | 0.93 | 0.31-2.80 | 0.90 |
| Hemoglobin | 1.40 | 0.65-2.99 | 0.39 | - | - | - |
| Myositis yes/no | 0.80 | 0.11-6.12 | 0.83 | - | - | - |
| ACA | **3.76** | **1.36-10.35** | **0.01** | **4.28** | **1.08-16.95** | **0.04** |
| ATA | 0.20 | 0.03-1.46 | 0.11 | 0.26 | 0.02-2.62 | 0.23 |
| Proton pump inhibitor | **3.07** | **1.28-7.49** | **0.01** | 2.90 | 0.96-8.75 | 0.06 |
| H2 receptor blocker | 2.39 | 1.01-5.72 | 0.05 | 1.60 | 0.55-4.69 | 0.39 |
| ACE inhibitor | 1.77 | 0.68-4.64 | 0.25 | - | - | - |
| Calcium channel blocker | 0.89 | 0.35-2.17 | 0.76 | - | - | - |
| Immunosuppressiva combined | 1,11 | 0.37-3.33 | 0.86 | - | - | - |
| Prostacyclin & ET-1 inhibitor | 2.23 | 1.11-8.7 | 0.04 | 1.59 | 0.48-6.01 | 0.48 |
| *Disease subset: entered as ordinal variable with order: non-cutaneous, limited and diffuse subset. # disease severity is a compound variable which included: ILD with FVC<70%, PAH, renal crisis, mRSS>20 or presence of digital ulcers. Medications are entered as yes or no, with a yes if the medication was used in the same year as the GIT questionnaire was completed. * Significant after Holm-Bonferroni correction. | | | | | | |

| **Table S8.**  Logistic regression analyses of baseline characteristics associated with moderate or severe emotional wellbeing symptom score in the 834 systemic sclerosis patients from the Leiden and Oslo cohort | | | | | | |
| --- | --- | --- | --- | --- | --- | --- |
|  | Univariable | | | Multivariable | | |
|  | **Moderate or severe emotional wellbeing symptom score** | | | | | |
|  | **OR** | **95% for CI OR** | **Significance P** | **OR** | **95% for CI OR** | **Significance P** |
| Female | **1.94** | **1.15-3.27** | **0.01** | **1.98** | **1.08-3.64** | **0.03** |
| Age, Years | 1.04 | 0.99-1.02 | 0.43 | 0.99 | 0.98-1.01 | 0.66 |
| Disease duration, years | **1.03** | **1.01-1.05** | **0.02** | **1.04** | **1.02-1.06** | **<0.001*** |
| Raynaud duration, years | **1.01** | **1.00-1.04** | **0.03** |  |  |  |
| Smoking ever | **1.68** | **1.15-2.46** | **0.007*** | **1.87** | **1.25-2.79** | **<0.001*** |
| Disease subset* | 1.04 | 0.58-1.86 | 0.90 | 1.71 | 0.80-3.67 | 0.17 |
| Disease severity | 1,28 | 0.91-1.82 | 0.16 | 1.12 | 0.74-1.70 | 0.59 |
| Weight loss (>10% in 1 year) | 1.13 | 0.63-2.06 | 0.68 | - | - | - |
| Hemoglobin | 1.09 | 0.81-1.47 | 0.55 | - | - | - |
| Myositis yes/no | 1.03 | 0.51-2.10 | 0.93 | - | - | - |
| ACA | **1.57** | **1.11-2.21** | **0.010** | 1.60 | 0.99-2.56 | 0.05 |
| ATA | **0.55** | **0.34-0.90** | **0.02** | 0.72 | 0.40-1.32 | 0.29 |
| Anti-RNApIII | 1.01 | 0.53-1.94 | 0.97 | - | - | - |
| Proton pump inhibitor | 1.17 | 0.83-1.67 | 0.37 | - | - | - |
| H2 receptor blocker | 0.83 | 0.56-1.22 | 0.33 | - | - | - |
| ACE inhibitor | 0.60 | 0.37-0.99 | 0.05 | 0.63 | 0.40-1.05 | 0.11 |
| Calcium channel blocker | 0.91 | 0.64-1.30 | 0.60 | - | - | - |
| Immunosuppressiva combined | 0.80 | 0.49-1.27 | 0.33 | - | - | - |
| Prostacyclin & ET-1 inhibitor | 1.35 | 0.66-2.33 | 0.63 | - | - | - |
| *Disease subset: entered as ordinal variable with order: non-cutaneous, limited and diffuse subset. # disease severity is a compound variable which included: ILD with FVC<70%, PAH, renal crisis, mRSS>20 or presence of digital ulcers. Medications are entered as yes or no, with a yes if the medication was used in the same year as the GIT questionnaire was completed. * Significant after Holm-Bonferroni correction. | | | | | | |

| **Table S9.**  Logistic regression analyses of baseline characteristics associated with moderate or severe social functioning symptom score in the 834 systemic sclerosis patients from the Leiden and Oslo cohort | | | | | | |
| --- | --- | --- | --- | --- | --- | --- |
| Univariable Multivariable | | | | | | |
| **Moderate or severe social functioning symptom score** | | | | | | |
|  | **OR** | **95% for CI OR** | **Significance**  **P** | **OR** | **95% for CI OR** | **Significance P** |
| Female | 1.57 | 0.95-2.58 | 0.08 | 1.45 | 0.83-2.54 | 0.19 |
| Age, Years | 1.00 | 0.99-1.01 | 0.95 | 0.99 | 0.98-1.01 | 0.35 |
| Disease duration, years | **1.03** | **1.01-1.05** | **<0.001*** | **1.04** | **1.01-1.06** | **<0.001*** |
| Raynaud duration, years | **1.01** | **1.01-1.07** | **0.01** |  |  |  |
| Smoking ever | **1.48** | **1.02-2.15** | **0.04** | **1.65** | **1.12-2.45** | **0.01** |
| Disease subset* | 1.50 | 0.80-2.79 | 0.21 | **2.27** | **1.03-5.02** | **0.04** |
| Disease severity | 1.10 | 0.78-1.58 | 0.56 | 1.02 | 0.67-1.55 | 0.92 |
| Weight loss (>10% in 1 year) | 1.12 | 0.70-1.81 | 0.63 | - | - | - |
| Hemoglobin | 1.01 | 0.76-1.35 | 0.94 | - | - | - |
| Myositis yes/no | 1.04 | 0.51-2.11 | 0.92 | - | - | - |
| ACA | **1.83** | **1.25-2.14** | **<0.001*** | **1.91** | **1.18-3.09** | **0.01** |
| ATA | 0.62 | 0.38-0.99 | 0.05 | 0.87 | 0.49-1.56 | 0.64 |
| Anti-RNApIII | 0.59 | 0.27-1.28 | 0.17 | - | - | - |
| Proton pump inhibitor | 1.36 | 0.96-1.93 | 0.08 | 1.33 | 0.89-1.87 | 0.12 |
| H2 receptor blocker | 0.88 | 0.60-1.30 | 0.53 | - | - | - |
| ACE inhibitor | 0.94 | 0.60-1.46 | 0.77 | - | - | - |
| Calcium channel blocker | 1.12 | 0.85-1.48 | 0.41 | - | - | - |
| MMF | 1.43 | 0.71-2.90 | 0.31 | - | - | - |
| Immunosuppressiva combined | 1.29 | 0.84-1.99 | 0.25 |  |  |  |
| Prostacyclin & ET-1 inhibitor | 1.88 | 0.88-2.67 | 0.33 | - | - | - |
| *Disease subset: entered as ordinal variable with order: non-cutaneous, limited and diffuse subset. # disease severity is a compound variable which included: ILD with FVC<70%, PAH, renal crisis, mRSS>20 or presence of digital ulcers. Medications are entered as yes or no, with a yes if the medication was used in the same year as the GIT questionnaire was completed. * Significant after Holm-Bonferroni correction.   \| **Table S10. Linear mixed model total cohort for total GIT symptom severity** \| \| \| \| \| \| \| \| --- \| --- \| --- \| --- \| --- \| --- \| --- \| \|  \| ***Univariable*** \| \| \| **Multivariable** \| \| \| \| **Predictor variable** \| **Coefficient** \| **95% CI** \| ***p* value** \| **Coefficient** \| **95% CI** \| **P value** \| \| Time * \|  \|  \|  \|  \|  \|  \| \| Sex \| 0.02 \| -0.005- -0.04 \| 0.01 \|  \|  \|  \| \| Age \| 0.005 \| -0.07-0.08 \| 0.14 \|  \|  \|  \| \| Disease duration \| 0.03 \| 0.002-0.05 \| 0.04 \|  \|  \|  \| \| Smoking \| -0.002 \| -0.03-0.03 \| 0.92 \|  \|  \|  \| \| Anti-centromere \| 0.13 \| 0.05-0.21 \| 0.002 \|  \|  \|  \| \| Anti-topoisomerase \| -0.09 \| -0.18-0.006 \| 0.07 \|  \|  \|  \| \| Skin involvement \| 0.01 \| -0.01-0.03 \| 0.28 \|  \|  \|  \| \| Disease subset \| 0.009 \| -0.01-0.03 \| 0.45 \|  \|  \|  \| \| ESR \| 0.02 \| -0.0004-0.04 \| 0.05 \|  \|  \|  \| \| Myositis \| 0.02 \| -0.002-0.04 \| 0.08 \|  \|  \|  \| \| PAH \| 0.02 \| -0.004-0.04 \| 0.12 \|  \|  \|  \| \| ILD \| 0.01 \| -0.01-0.04 \| 0.25 \|  \|  \|  \| \| ACE inhibitor \| 0.01 \| -0.01-0.04 \| 0.32 \| 0.01 \| -0.01-0.04 \| 0.35 \| \| CCB \| -0.002 \| -0.03-0.03 \| 0.87 \| -0.002 \| -0.03-0.03 \| 0.89 \| \| PPI \| 0.01 \| -0.02-0.04 \| 0.44 \| 0.009 \| -0.02-0.04 \| 0.53 \| \| ET-1 inhibitor \| 0.02 \| -0.003-0.04 \| 0.10 \| 0.02 \| -0.003-0.04 \| 0.10 \| \| H2 blocker \| 0.02 \| -0.007-0.05 \| 0.15 \| 0.02 \| -0.007-0.05 \| 0.16 \| \| Corticosteroids \| 0.02 \| -0.007-0.04 \| 0.18 \| 0.02 \| -0.007-0.04 \| 0.19 \| \| Methotrexate \| 0.02 \| 0.0001-0.04 \| 0.05 \| 0.02 \| -0.005-0.04 \| 0.12 \| \| Azathioprine \| 0.02 \| - 0.03-0.04 \| 0.22 \| 0.02 \| -0.003-0.04 \| 0.33 \| \| Hydroxychloroquine \| 0.02 \| - 0.003-0.04 \| 0.33 \| 0.02 \| -0.003-0.04 \| 0.25 \| \| MMF \| 0.02 \| - 0.0002-0.04 \| 0.52 \| 0.02 \| -0.0002-0.04 \| 0.54 \| \| Cyclophosphamide \| 0.01 \| -0.01-0.03 \| 0.33 \| 0.01 \| -0.01-0.03 \| 0.34 \| \| Coefficients shown are the time coefficients. Separate multivariable regression analyses with adjustment for time, age, sex and ACA. ILD= interstitial lung disease, PAH= pulmonary arterial hypertension, mRSS= modified Rodnan Skin Score, CCB= calcium channel blockers, PPI = proton pump inhibitors, ET-1= endothelin receptor antagonist, ACE= Angiotensin-converting-enzyme inhibitors, ESR= erythrocyte sedimentation rate, MMF= Mycophenolate mofetil. \| \| \| \| \| \| \| | | | | | | |


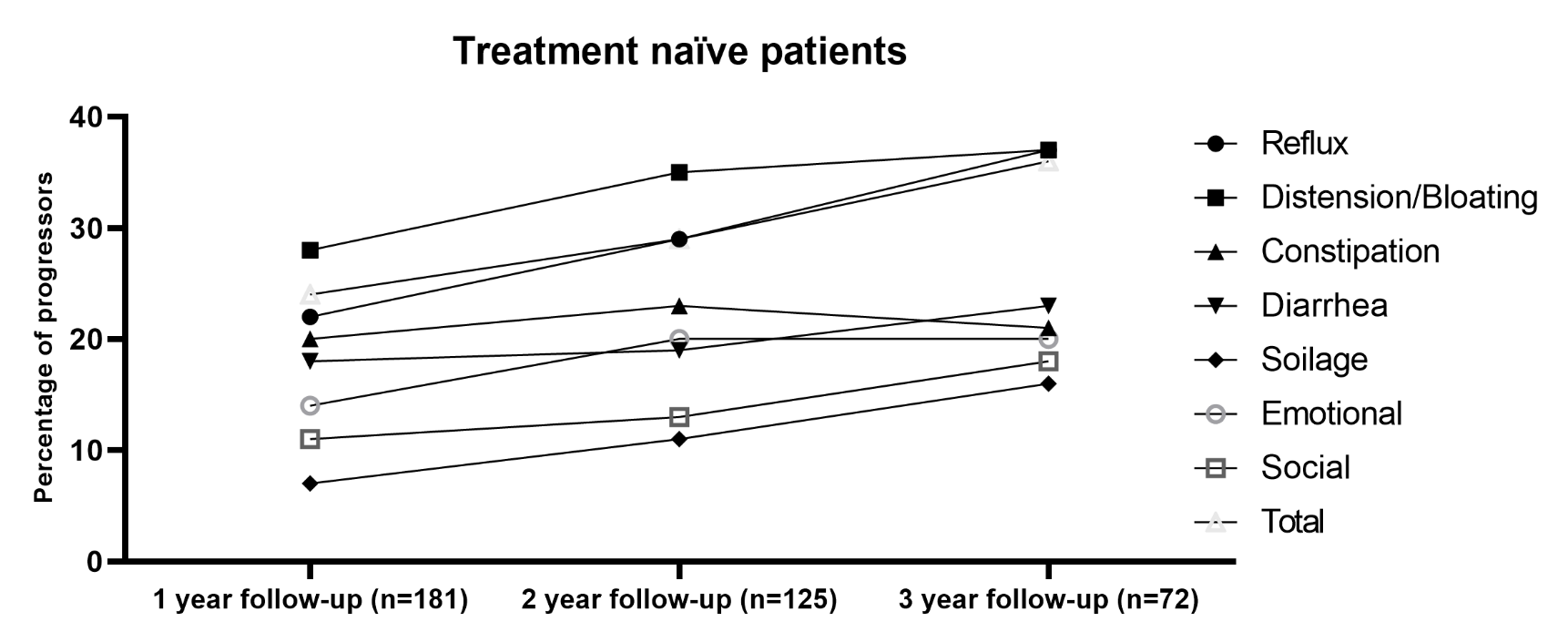


Figure S1. Percentage of progressors per GIT subdomain over the follow-up period in treatment naïve patients in the inception cohort (for immunosuppressive treatment). The lines indicate years of follow-up and the numbers (n) the amount of treatment naïve patients at that time point. We calculated the percentage of progressors based on the MCID of the UCLA GIT 2.0 per subdomain after 1,2 and 3 years of follow-up.

1. Khanna D, Hays RD, Maranian P, Seibold JR, Impens A, Mayes MD, et al. Reliability and validity of the University of California, Los Angeles Scleroderma Clinical Trial Consortium Gastrointestinal Tract Instrument. Arthritis and rheumatism. 2009;61(9):1257-63.

2. Khanna D, Furst DE, Maranian P, Seibold JR, Impens A, Mayes MD, et al. Minimally important differences of the UCLA Scleroderma Clinical Trial Consortium Gastrointestinal Tract Instrument. J Rheumatol. 2011;38(9):1920-4.

3. McMahan ZH, Paik JJ, Wigley FM, Hummers LK. Determining the Risk Factors and Clinical Features Associated With Severe Gastrointestinal Dysmotility in Systemic Sclerosis. Arthritis Care Res (Hoboken). 2018;70(9):1385-92.

4. Baron M, Hudson M, Steele R. Malnutrition is common in systemic sclerosis: results from the Canadian scleroderma research group database. J Rheumatol. 2009;36(12):2737-43.

5. Garros A, Marjoux S, Khouatra C, Coppere B, Grange C, Hot A, et al. Prevalence of fecal incontinence in a cohort of systemic sclerosis patients within a regional referral network. United European gastroenterology journal. 2017;5(7):1046-50.

6. Hudson M, Lo E, Lu Y, Hercz D, Baron M, Steele R. Cigarette smoking in patients with systemic sclerosis. Arthritis and rheumatism. 2011;63(1):230-8.

7. Jaovisidha K, Csuka ME, Almagro UA, Soergel KH. Severe gastrointestinal involvement in systemic sclerosis: report of five cases and review of the literature. Seminars in arthritis and rheumatism. 2005;34(4):689-702.

8. Richard N, Hudson M, Wang M, Gyger G, Proudman S, Stevens W, et al. Severe gastrointestinal disease in very early systemic sclerosis is associated with early mortality. Rheumatology (Oxford, England). 2019;58(4):636-44.

9. Kuribayashi S, Motegi SI, Hara K, Shimoyama Y, Hosaka H, Sekiguchi A, et al. Relationship between esophageal motility abnormalities and skin or lung involvements in patients with systemic sclerosis. Journal of gastroenterology. 2019.

10. Richardson C, Agrawal R, Lee J, Almagor O, Nelson R, Varga J, et al. Esophageal dilatation and interstitial lung disease in systemic sclerosis: A cross-sectional study. Seminars in arthritis and rheumatism. 2016;46(1):109-14.

11. Furst DE, Clements PJ, Steen VD, Medsger TA, Jr., Masi AT, D'Angelo WA, et al. The modified Rodnan skin score is an accurate reflection of skin biopsy thickness in systemic sclerosis. J Rheumatol. 1998;25(1):84-8.
